# Lateral Vibration Control of Long-Span Small-Radius Curved Steel Box Girder Pedestrian Bridge with Distributed Multiple Tuned Mass Dampers

**DOI:** 10.3390/s22124329

**Published:** 2022-06-07

**Authors:** Zhaolan Wei, Mengting Lv, Siyin Wu, Minghui Shen, Meng Yan, Shaomin Jia, Yi Bao, Peng Han, Zuyin Zou

**Affiliations:** 1 School of Civil Engineering, Sichuan Agricultural University, Dujiangyan 611830, China; 2020326036@stu.sicau.edu.cn (M.L.); 2019326005@stu.sicau.edu.cn (S.W.); 2021326038@stu.sicau.edu.cn (M.S.); 41480@sicau.edu.cn (S.J.); 2 Department of Bridge Engineering, Southwest Jiaotong University, Chengdu 610031, China; yanmeng@jdjc.net; 3 Department of Civil, Environmental and Ocean Engineering, Stevens Institute of Technology, Hoboken, NJ 07030, USA; yi.bao@stevens.edu; 4 Latin American Department, SINOMACH-HI International Equipment Co., Ltd., Beijing 100176, China; gabrieldachina@126.com

**Keywords:** bridge vibration, curved bridge, distributed multiple tuned mass dampers (MTMD), lateral vibration, pedestrian bridge, steel box girder

## Abstract

Curved pedestrian bridges are important urban infrastructure with the desired adaptability to the landscape constraints and with aesthetic benefits. Pedestrian bridges feature thin cross-sections, which provide sufficient load capacities but lead to low natural frequencies that make the bridges susceptible to vibration under pedestrian excitation. This study investigates the lateral vibration of a curved bridge with a small radius down to 20 m, proposes an approach to mitigate the lateral vibration of bridges with large curvatures using distributed multiple tuned mass dampers (MTMD), and conducts in-situ bridge tests to evaluate the vibration mitigation performance. The lateral vibration was investigated through in-situ tests and finite element analysis as well as the code requirements. The key parameters of the distributed MTMD system were improved by strategically selecting the mass ratio, bandwidth, center frequency ratio, and damper number. The results showed that the curved bridge was subjected to significant lateral vibration due to the coupling of torque and moment, and the recommended design parameters for the studied bridge were derived, i.e., the total mass ratio is 0.02, bandwidth is 0.15, center frequency ratio is 1.0, and damper number is 3. The proposed approach effectively improves the deployment of MTMD for lateral vibration control of the curved bridge. The field tests showed that the vibration was reduced by up to 82% by using the proposed approach.

## 1. Introduction

Pedestrian bridges play important roles in improving mobility through diverging pedestrians and vehicles, improving pedestrian safety, and mitigating congestion, particularly in major cities. With the shrinkage of available spaces in the course of urbanization, curved pedestrian bridges attract intensive interest because of their adaptability to the landscape constraints and aesthetic advantages in urban infrastructure [1]. The span lengths of curved pedestrian bridges keep increasing, and thin cross-sections have been adopted in many curved bridges. The adoption of thin cross-sections makes curved bridges aesthetically appealing. However, the use of thin sections led to low flexural and torsional stiffness as well as low natural frequencies of the bridges [2]. The fundamental frequencies of many pedestrian bridges are lower than 3 Hz [3], which are comparable with the frequency of pedestrian excitation. Pedestrian loads may cause resonance of the bridges and lead to catastrophic consequences such as collapse [4]. The vibration of bridges compromises their serviceability and durability [5].

An effective method to mitigate the vibration problems for curved pedestrian bridges is to employ dampers [6,7,8,9,10,11]. Bar hysteretic dampers were proposed to dissipate energy through bending mechanisms [10,11], and shape memory alloy was used to replace steel bars to achieve re-centering properties [11]. Among different types of dampers, tuned mass dampers were adopted for vibration control in many projects since they are cost-effective and easy to deploy in both new and existing structures [12,13,14]. Employment of tuned mass dampers mitigated resonance at specific frequencies. Based on tuned mass dampers, multiple tuned mass dampers (MTMD) were proposed and reduced the vibration of complex structures such as high-rise buildings and long-span bridges [15,16,17], since the MTMD mitigated resonance at different frequencies. Then, MTMD was utilized in vibration control for various engineering structures under operation loads and extreme events [18,19,20,21]. Previous research showed that the deployment positions and parameters of MTMD played significant roles in the performance of vibration control [22,23,24,25,26].

MTMD have been applied to pedestrian bridges for vibration control. For example, a single-span 34-m-long girder bridge was investigated [27]. The fundamental natural frequency of the bridge was 2.0 Hz. The use of MTMD reduced the maximum acceleration from 1.3 m/s^2^ to 0.4 m/s^2^. MTMD were applied to a 40-m-long girder bridge [28]. The fundamental natural frequency of the bridge was 2.0 Hz. The use of MTMD reduced the maximum acceleration from 2.5 m/s^2^ to 0.2 m/s^2^. MTMD were also applied to a 60-m-long girder bridge [29]. The fundamental natural frequency of the bridge was 1.33 Hz. The use of MTMD reduced the maximum acceleration from 15.3 m/s^2^ to 1.35 m/s^2^. An eight-span 67-m-long girder bridge was investigated [30]. The fundamental natural frequency of the bridge was 2.94 Hz. The use of MTMD reduced the maximum acceleration from 1.6 m/s^2^ to 0.3 m/s^2^. Wang and Shi [31] applied MTMD to a single-span 55.2-m-long girder bridge. The fundamental natural frequency of the bridge was 2.55 Hz. The use of MTMD reduced the maximum acceleration from 0.75 m/s^2^ to 0.15 m/s^2^. Recently, MTMD were applied to suspension and cable-stayed pedestrian bridges [32,33].

The previous research showed that MTMD reduced bridge vibration. However, the previous research mainly focused on the vertical vibration of bridges with straight girders. There is limited research on the lateral vibration of curved pedestrian bridges. Another limitation is that the radius of the studied curved pedestrian bridges is large (≥50 m). As the radius decreases, the coupling effect of torsion and flexure is highly magnified and exacerbates lateral vibration of curved bridges. There is a lack of knowledge about the effect of MTMD on the lateral vibration of curved pedestrian bridges with large curvatures. It is unclear how much vibration amplitude can be reduced and how the placement and parameters of MTMD should be improved. To address these problems, this research investigates the performance of MTMD for lateral vibration control in a curved pedestrian bridge with a center radius down to 20 m. This research has three main objectives: (1) to study the lateral vibration of the curved pedestrian bridge under pedestrian loads; (2) to understand the effect of MTMD on the lateral vibration, and; (3) to study the influences of the key parameters of MTMD for strategical deployment in vibration control.

To achieve these objectives, in-situ tests and three-dimensional finite element analysis of the bridge were performed to investigate the lateral vibration of the bridge under pedestrian loads. A parametric study was performed to evaluate the effects of key parameters of the MTMD. The investigated parameters included the mass ratio, bandwidth, center frequency ratio, and damper number. Based on the parametric study, the distributed MTMD were strategically configured for vibration control.

The significance of this research includes three main aspects: (1) This research offers knowledge on lateral vibration and vibration control for curved bridges with large curvatures. (2) This research clarifies the effects of key variables of MTMD systems and provides guidelines for the strategic selection of the variables. (3) The research outcomes advance the applications of MTMD for the safe and comfortable operation of pedestrian bridges.

## 2. Vibration Investigations

### 2.1. Bridge Description

**Figure 1:**
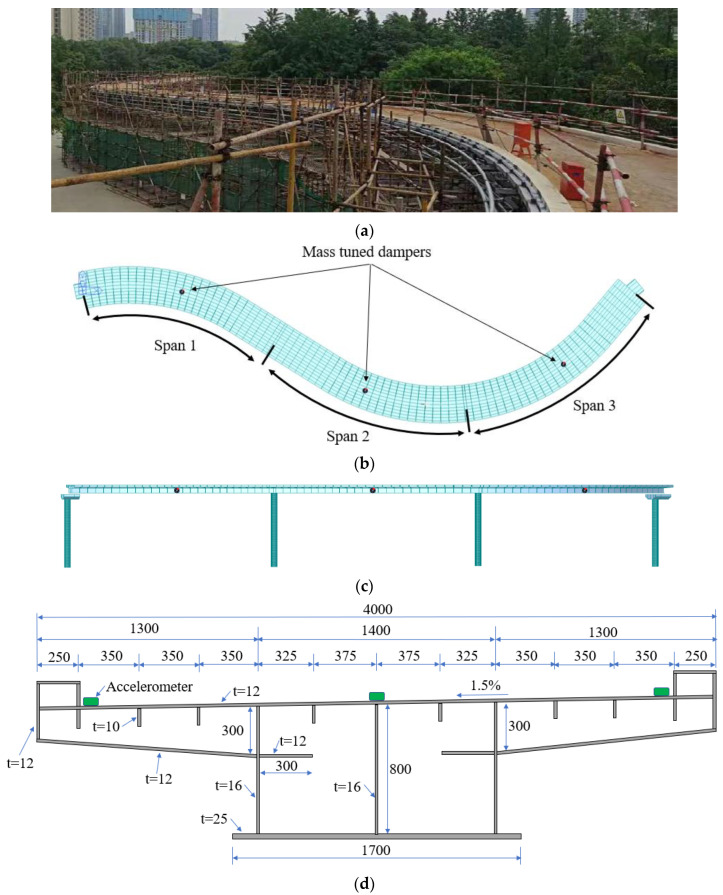
Illustration of the investigated bridge (unit: mm): (**a**) a photo; (**b**) the plan view; (**c**) the elevation view; and (**d**) the cross-section of the steel box girder.

The investigated bridge is a 3-span 60-m-long pedestrian bridge with curved steel box girders. The length of each span is 20 m. The width and depth of the steel box girders are 4.0 m and 0.8 m, respectively. The center radius of the curved steel box girders was 20 m. The geometry and cross-section of the girders of the bridge are shown in Figure 1.

### 2.2. In-Situ Tests

**Figure 2:**
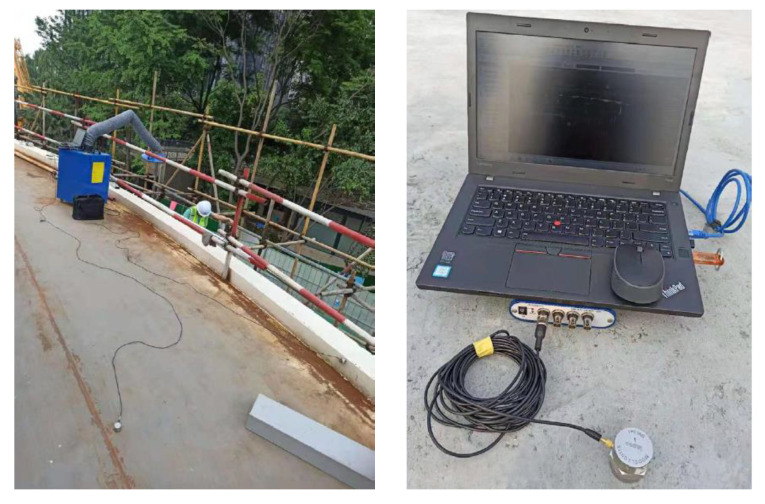
Instrumentation of the investigated pedestrian bridge with accelerometers.

The three spans of the bridge were respectively tested after the bridge was erected and instrumented with accelerometers. Nine accelerometers were deployed in the bridge that had three spans. In each span, three accelerometers were deployed at the mid-span section, as depicted in Figure 1d. Along the transverse (width) direction of the bridge, the three accelerometers were attached to the bridge deck in the center and at the two sides of the mid-span section, as shown in Figure 2.

The accelerometers (model: LC0116) measured the accelerations of the bridge along its longitudinal (length), transverse (width), and vertical directions. The acceleration data from the accelerometers were collected using a data acquisition system (model: INV3062). For each span of the bridge, ambient vibration tests were carried out to evaluate the natural frequencies of the pedestrian bridge. The bridge was excited by vehicles passing underneath the bridge and the winds. Accelerations were measured from the accelerometers for an extended time longer than 600 s, which is more than 400 times the fundamental period of the bridge. The sampling frequency was 45 Hz. The measured acceleration history data were processed using a filter with a passing bandwidth from 0.5 Hz to 45 Hz [32]. The first five characteristic frequencies were directly identified from the power spectrum, which are 0.776 Hz, 1.100 Hz, 1.335 Hz, 1.992 Hz, and 2.732 Hz.

### 2.3. Finite Element Analysis

A three-dimensional finite element model was established for further analysis of the bridge using a commercial software MIDAS CIVIL, which is a well-developed software used to analyze the static and dynamic responses of bridges, as elaborated in references [34,35]. The curved steel box girder was simulated using three-dimensional two-node beam elements. The cross-sections of the beam elements were consistent with the steel box girder of the real bridge. The cap girders and piers were also simulated using beam elements, and the cross-sections of the beam elements were consistent with the geometry of the real bridge. A total of 372 beam elements were created.

**Figure 3:**
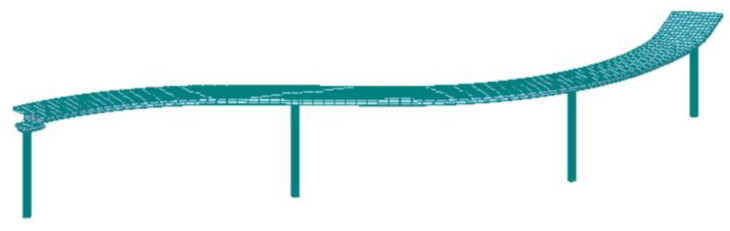
The established three-dimensional finite element model of the investigated bridge.

The connection between the box girder and cap girders was defined using spring elements with six degrees of freedom that were used to define the rigidity of the supporting conditions. Along the vertical and the transverse (bridge width) directions, the stiffness of the bearings was assumed to be infinite. Along the longitudinal (bridge length) direction, the stiffness was assumed to be zero, meaning that the box girders were simply supported by the cap girders of the piers. Figure 3 shows the finite element model.

The finite element model was used to analyze the natural frequencies and mode shapes of the bridge through eigenvalue analysis. The analysis results of the natural frequencies and the mode shapes of the first five modes are listed in Table 1.

The first two modes were lateral bending modes, and the frequencies were low, indicating low lateral stiffness. The natural frequencies of the first five modes were lower than 3 Hz, comparable with the frequency of pedestrian loads. The analysis results of natural frequencies were compared with measurement results from in-situ tests. The maximum discrepancy between measurement and simulation results was 5%, indicating that the finite element model provided adequate accuracy in the prediction of vibration characteristics.

### 2.4. Pedestrian Loads and Investigation Cases

The pedestrian loads recommended by ISO specifications were adopted in this research because the recommended loads covered vertical and lateral loads for single pedestrian and multiple pedestrian scenarios [36,37] The pedestrian loads are treated as cyclic loads expressed as harmonic waves as shown in Equation (1):

(1) $$F\left(t\right)=G\left[\overset{k}{\underset{i=1}{\sum }}\alpha_{i,h}sin(2\pi if_{s}t+\varphi_{i,h})\right]$$

where *G* is the average weight of a person (*G* = 750 *n*); *α_i_* is the dynamic factor of excitation of the *i*-th order (*α*_1_ = 0.4 for vertical pedestrian loads, and *α*_1_ = 0.1 for transverse loads); *f_s_* is the pedestrian pace frequency (unit: Hz); *t* is time (unit: s); and *φ_i_* is the phase angle of excitation of the *i*-th order.

When there is a single pedestrian on the bridge, the vertical and transverse pedestrian loads are respectively expressed by Equations (2) and (3):

(2) $$F_{v}\left(t\right)=0.4\times 750sin(2\pi f_{s}t),$$

(3) $$F_{h}\left(t\right)=0.1\times 750sin(2\pi f_{s}t),$$

The crowd load is expressed by Equation (4):

(4) $$F{\left(t\right)}_{n}=nF\left(t\right)C\left(n\right)$$

where *n* is the equivalent number of pedestrians; C(n) is a factor used to account for interactions between pedestrians, which can be calculated by [36,37]:

(5) $$C\left(n\right)=\sqrt{n}/n$$

(6) n=WLS

where *W*, *L*, and *S* are the bridge width, bridge length, and crowd density (*S* = 0.6 person/m^2^), respectively.

Table 2 lists six pedestrian load cases, including three cases for single pedestrian load and three cases for crowd load. For the cases of single pedestrian load, three pace frequencies were considered, which are 0.739 Hz, 1.800 Hz, and 2.300 Hz, representing the first-order resonant frequency of the bridge, normal walking, and quick walking, respectively. The single pedestrian load was applied as a concentrated force to the mid-span sections of each span of the bridge. For the crowd load, the same pace frequencies were considered. The crowd load was applied as a line force to each span of the bridge.

**Figure 4:**
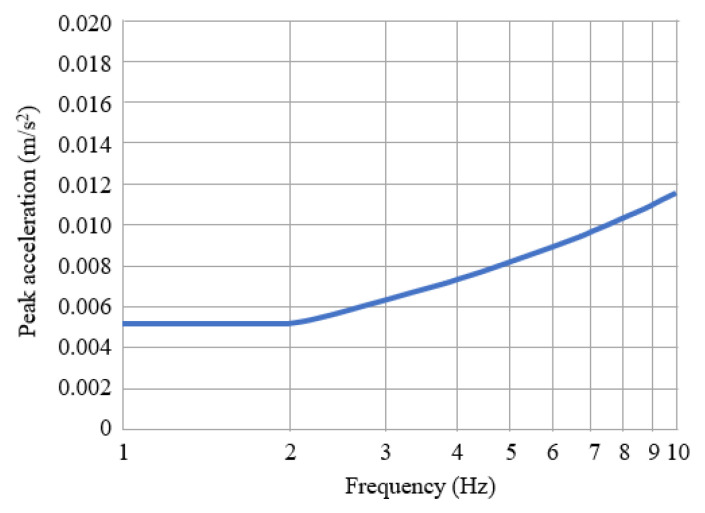
The threshold of lateral accelerations at different frequencies as specified by ISO.

The serviceability of the bridge was evaluated according to the ISO specifications [36,37,38]. The threshold of accelerations was limited at each frequency, as shown in Figure 4. The accelerations of pedestrian bridges should be equal to or lower than the threshold. The ISO specifications were applied to both the vertical and transverse directions of the bridge.

### 2.5. Evaluation of Vibration Characteristics

The finite element model was used to analyze the vibration characteristics of the bridge in the six load cases. In each case, the accelerations of different bridge sections were determined, as listed in Table 3. The listed results include the root mean square and maximum values of lateral accelerations of the mid-span (1/2) sections of the first and third spans as well as the quarter spans (1/4, 1/2, and 3/4) of the second span.

The comparison of the analysis results of Cases 1 to 3 for single pedestrian loads revealed that the single pedestrian loads had limited effects on the lateral accelerations at the different sections of the bridge. The limits of the lateral acceleration were 0.15–0.16 m/s^2^, while the maximum lateral acceleration was lower than 0.02 m/s^2^. The change of the pace frequency did not significantly change the lateral accelerations. In comparison, Cases 4 to 6 for crowd pedestrian loads generated more intensive lateral accelerations than the single pedestrian load cases, and the pace frequency showed significant effects. The limits of the lateral accelerations were 0.30–0.32 m/s^2^, while the maximum lateral acceleration was lower than 0.345 m/s^2^. The maximum lateral acceleration occurred at the middle span of the third span, which exceeded the code limit (0.3 m/s^2^) of the ISO specification [36,37].

**Figure 5:**
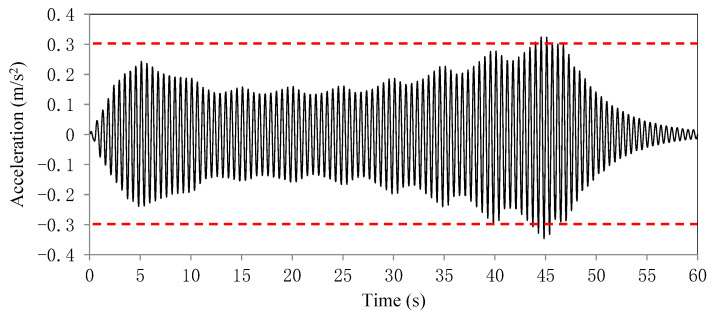
Plot of the time history result of the mid-span lateral acceleration of the third span of the investigated bridge (unit: m/s^2^).

The time history curve of the mid-span lateral acceleration of the third span is plotted in Figure 5. The dash lines mark the code limit value. The curve shows that the vibration amplitude increased with time overall until the crowd loads left the bridge.

## 3. Vibration Mitigation

### 3.1. Tuned Mass Dampers

**Figure 6:**
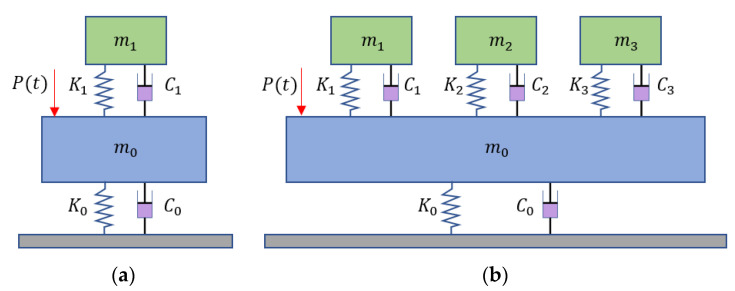
Comparison of the mechanical models of: (**a**) single tuned mass damper (STMD) and (**b**) multiple tuned mass dampers (MTMD).

Previous research showed that MTMD were effective in mitigating the vertical vibration of bridges. It is speculated that MTMD are also effective in controlling the lateral vibration of curved pedestrian bridges. To test this speculation, the finite element model was modified to incorporate tuned mass dampers. Both single tuned mass damper (STMD) and MTMD were considered. The mechanical models of STMD and MTMD are illustrated in Figure 6.

The main difference between STMD and MTMD is that MTMD have multiple damper masses attached to the bridge. The multiple dampers are deployed at different positions of the bridge. For each damper, the key parameters include the mass, stiffness, and damping ratio [39]. When a STMD is adopted, the optimal frequency and damping ratios can be calculated as:

(7) $$f_{1}=\frac{1}{1\;+\;\mu },$$

(8) $$\zeta_{1}=\sqrt{\frac{3\mu }{8{\left(1\;+\;\mu \right)}^{3}}},$$

(9) $$\mu =\frac{m_{1}}{m_{0}},$$

where *f*_1_ is the optimal center frequency ratio; *ζ*_1_ is the optimal damping ratio, and; *μ* is the mass ratio.

Then, the optimal stiffness (*k*_1) and damping (*c*_1) of the STMD are calculated as:

(10) $$k_{1}=f_{1}{}^{2}\omega_{0}{}^{2}m_{0},$$

(11) $$c_{1}=2\zeta_{1}f_{1}\omega_{0}m_{0},$$

where *ω*_0_ is the natural frequency of the bridge and *m*_0_ is the transverse modal mass of the bridge. With the above formulae, the parameters of the STMD were determined. The mass was 1973.6 kg. The stiffness was 242.4 kN/m. The damping ratio was 1.84 kN·s/m. The natural frequency was 1.8 Hz.

When MTMD are used, the design parameters include the mass ratio, stiffness, bandwidth, damping ratio, and damper number. According to reference [40], the main design parameters can be calculated as:

(12) $$\omega_{j}=\omega_{T}\left[1\;+\;\left(j\;-\;\frac{n\;+\;1}{2}\right)\frac{R}{n\;-\;1}\right],$$

(13) $$k_{j}=\frac{\mu m_{0}}{\sum_{j=1}^{n}1/{\left(2\pi \omega_{j}\right)}^{2}}$$

(14) $$c_{j}=2\zeta_{j}k_{j}/\left(2\pi \omega_{j}\right)$$

(15) $$m_{j}=k_{j}/{\left(2\pi \omega_{j}\right)}^{2},$$

where *ω_T_* is the average natural frequency of *n* TMDs; *ω_j_*, *k_j_*, *c_j_*, and *ζ_j_* are the natural frequency, stiffness, damping, and damping ratio of the *j*-th damper, and; *R* is the bandwidth.

Three tuned mass dampers were placed in the three spans of the bridge, designated as TMD-1 to TMD-3, respectively. The total mass ratio was 0.02. The bandwidth was 0.2. The damping ratio was 0.016. Then, the design parameters of the MTMD were calculated. The mass values of TMD-1 to TMD-3 were 795.0 kg, 644.6 kg, and 532.8 kg, respectively. The stiffness, damping ratio, and natural frequencies of TMD-1 to TMD-3 were 82.4 kN/m, 0.233 kN·s/m, and 1.8 Hz, respectively. The deployment positions of the three dampers are marked by red dots in Figure 1b.

With the parameters of STMD and MTMD, the tuned mass dampers were simulated using spring elements and node mass in the finite element model. More details of the modeling of the tuned mass dampers are elaborated on in references [38,40]. For the STMD, the damper was simulated using a spring element and a node mass at the mid-span section of Span 2 of the bridge. For the MTMD, three dampers were placed at the middle spans of the three spans. The lateral vibration results of the bridge under load case 5 are listed in Table 4.

The results indicated that the STMD and MTMD reduced the maximum lateral accelerations. When the STMD was used, the percentages of reduction were higher than 34%. At the middle span of the third span, the maximum lateral acceleration was reduced from 0.345 m/s^2^ to 0.229 m/s^2^, satisfying the code requirement. When the MTMD were used, the percentages of reduction were higher than 77%. At the mid-span section of the third span, the amplitude of lateral acceleration was reduced from 0.345 m/s^2^ to 0.059 m/s^2^, satisfying the code requirement. In comparison, the use of MTMD was more effective in reducing the lateral vibration of the bridge, and MTMD achieved comparable percentages of reduction at the different sections, while the STMD led to different percentages of reduction at the different sections.

### 3.2. Parametric Studies

Sensitive analysis is a method of analyzing system stability. It is assumed that there is a system whose characteristic P is mainly determined by multiple factors $a=\left\{a_{1},a_{2},\ldots ,a_{n}\right\}$. At a certain reference state $a^{*}=\left\{a_{1}^{*},a_{2}^{*},\ldots ,a_{n}^{*}\right\}$, the system characteristics are $P^{*}$, which makes each influencing factor vary within its own suitable range, causing the system with the characteristic P to deviate from the reference state $P^{*}$. This method is called sensitivity analysis. For the MTMD vibration damping system of the pedestrian bridge, the characteristic P is an acceleration response under the MTMD action. In this paper, the influencing factor a is taken as the total mass ratio μ, the frequency bandwidth ΔR, the center frequency ratio $x_{c}$, and TMD number *n*, so $P=f(\mu ,\Delta R,x_{c},n)$. According to research and engineering practical applications, μ is usually taken between 0.01 to 0.05; ΔR is usually about 0.15, and $x_{c}$ is usually about 1 or so. While *n* varies for different pedestrian bridges, it should not be too large for cost purposes.

Further research was performed to investigate the effects of four important parameters of MTMD, which were the total mass ratio, bandwidth, center frequency ratio, and damper number. The results of parametric studies were used to strategically configure the design of the MTMD for the curved pedestrian bridge. In the parametric studies, one parameter was evaluated each time.

To evaluate the effect of the total mass ratio, the other parameters were kept constant. The bandwidth was 0.15. The center frequency ratio was 1.0. The damper number was 3. Four total mass ratios were tested, which were 0.01, 0.02, 0.03, and 0.04. The three dampers were placed at the mid-span sections of the three spans. The stiffness of the three dampers was denoted by *k*_1_, *k*_2_, and *k*_3_, respectively. Then, the mass and stiffness of each damper can be calculated using Equations (12)–(15), as listed in Table 5. As the total mass ratio increased from 0.01 to 0.04, the optimal stiffness of each tuned mass damper of the MTMD linearly increased.

With the determined design parameters, the lateral accelerations at the five cross-sections of the bridge were analyzed using the finite element model incorporating the MTMD, as listed in Table 6. At all the five sections, the minimum lateral acceleration was achieved when the total mass ratio was 0.02, which was selected as the total mass ratio for further research.

In the evaluation of the effect of the bandwidth, the total mass ratio was kept at 0.02; the center frequency ratio was 1.0, and; the damper number was 3. Five bandwidths were tested, which were 0.05, 0.10, 0.15, 0.20, and 0.25. The three dampers were placed at the mid-span sections of the three spans. The stiffness of the three dampers was denoted by *k*_1_, *k*_2_, and *k*_3_, respectively. Then, the mass and stiffness of each damper of the MTMD were calculated using Equations (12)–(15), as listed in Table 7. As the bandwidth increased from 0.05 to 0.25, the optimal stiffness of each tuned mass damper of the MTMD slightly increased.

With the determined design parameters, the lateral accelerations at the five cross-sections of the bridge were analyzed using the finite element model incorporating the MTMD, as listed in Table 8. At all the five sections, the lateral acceleration achieved the minimum values when the bandwidth was 0.15, which was therefore selected as the bandwidth for further parametric studies.

In the evaluation of the effect of the center frequency ratio, the total mass ratio was 0.02; the bandwidth was 0.15, and; the number of dampers was 3. Five center frequency ratios were tested, which were 0.95, 0.98, 1.00, 1.02, and 1.05. The three dampers were placed at the mid-span sections of the three spans. The stiffness of the three dampers was denoted by *k*_1_, *k*_2_, and *k*_3_, respectively. Then, the mass and stiffness of each damper of the MTMD were calculated using Equations (12)–(15), as listed in Table 9. As the center frequency ratio increased from 0.95 to 1.05, the optimal stiffness of each tuned mass damper of the MTMD slightly increased.

With the determined design parameters, the lateral accelerations at the five cross-sections of the bridge were analyzed using the finite element model incorporating the MTMD, as listed in Table 10. At all the five sections, the lateral acceleration achieved the minimum values when the center frequency ratio was 1.00, which was therefore selected as the center frequency ratio for further parametric studies.

In the evaluation of the effect of the damper number, the total mass ratio was kept at 0.02; the bandwidth was 0.15, and; the center frequency ratio was 1.0. Four damper numbers were tested, which were 1, 3, 5, and 9. When one damper was used, the damper was deployed at the mid-span section of the second span of the bridge. When three dampers were used, they were deployed at the mid-span sections of the three spans. When five dampers were used, three dampers were deployed at the mid-span sections of the three spans, and two dampers were deployed at the quarter spans of the second span. When nine dampers were used, they were deployed at the 1/4, 1/2, and 3/4 spans of the three spans. The stiffness of the dampers was denoted by *k*_1_ to *k*_9_. Then, the mass and stiffness of each damper of the MTMD were calculated using Equations (12)–(15), as listed in Table 11. As the damper number increased from 1 to 9, the optimal stiffness of the individual dampers decreased.

With the determined design parameters, the lateral accelerations at the five cross-sections of the bridge were analyzed using the finite element model incorporating the MTMD, as listed in Table 12. At all the five sections, the lateral acceleration decreased with the increase of the damper number. A mean percentage of reduction was used to evaluate the effect of damper number on the reduction of lateral accelerations. The mean percentage of reduction is defined as the percentage of reduction divided by the damper number.

As the damper number increased from 3 to 9, although the lateral acceleration continued to decrease, the mean percentage of reduction decreased. To balance the vibration mitigation performance and cost of MTMD, the damper number was determined to be 3.

The studied design parameters exhibited significant effects on the performance of MTMD. With the investigated ranges of the parameters, the lateral acceleration varied in the range of 0.027–0.051 m/s^2^ for the mass ratios; 0.027–0.061 m/s^2^ for the bandwidths; 0.027–0.069 m/s^2^ for the frequency ratios, and; 0.027–0.229 m/s^2^ for the damper numbers. The comparison of these ranges indicated that the damper number had the highest effect.

## 4. Performance Evaluation

Based on the selected parameters of the MTMD system, in-situ tests were conducted to test the performance of the MTMD system. The adopted parameters were *μ* = 0.02, Δ*R* = 0.15, *λ* = 1, and *n* = 3. The three dampers were deployed in the three spans of the bridge. In each span, one damper was deployed at the mid-span section. The mass of each damper was 201.3 kg. The effect of the additional mass due to the use of the MTMD on the static stresses of the bridge was examined using the finite element model. The mechanical responses of the bridge satisfied the code requirements [37].

As described earlier, the mid-span sections of the three bridge spans were also instrumented with accelerometers, which were used to measure accelerations along the lateral (width) direction of the bridge. Two load cases were investigated, as listed in Table 13. The errors between the simulation and the test results of accelerations were mainly related to the assumptions of the finite element model: (1) The crowd loads applied by ten people in the real bridge were simplified in the finite element analysis. In real tests, the ten people were not ideally synchronized and had consistent pace frequency. These were not fully considered in the finite element model. (2) There were assumptions on the material properties and boundary conditions in the finite element analysis. Despite the errors, the simulation results were deemed valid because of the complexity of the tests.

**Figure 7:**
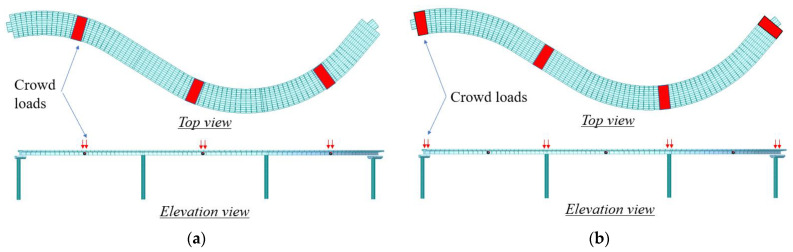
Depiction of the two load cases of in-situ tests of the bridge instrumented with three mass tuned dampers: (**a**) Case I and (**b**) Case II. A red area represents 10 adults, with an average weight of 750 *n* per person and distributed at 0.6 of a person per m^2^.

In Case I, ten people jumped at the mid-span sections of the three spans to excite the bridge vibration. In Case II, ten people jumped at the end-span sections of the three spans to excite the bridge vibration. The applied crowd loads of the two cases are depicted in Figure 7. Each of the red areas represents 10 adults. The average weight of a person was 750 *n*, which was distributed in an area of 0.6 m^2^. In both cases, the lateral accelerations of the bridge were respectively evaluated under the conditions of “without MTMD” and “with MTMD”.

It was noted that the MTMD system was installed in the bridge under both conditions in each of the load cases. The difference between the “without MTMD” and “with MTMD” conditions was that the MTMD system was locked under the “without MTMD” condition while the MTMD system was released under the “with MTMD” condition. When the MTMD system was locked, it acted as lumped masses and did not have a damping effect. When the MTMD system was released, the system acted as the dampers.

**Figure 8:**
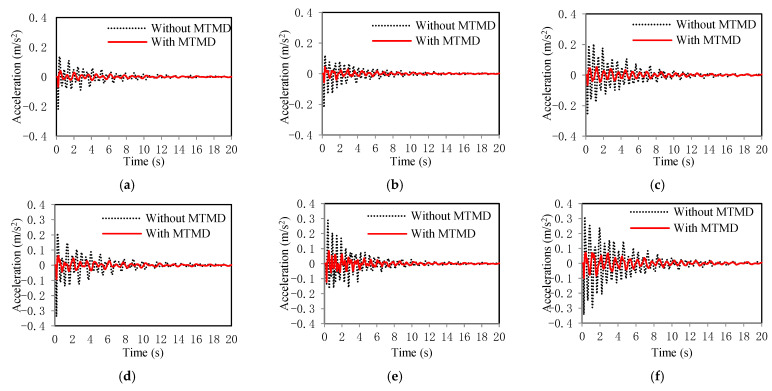
Results of lateral accelerations: (**a**) the first span in Case I; (**b**) the second span in Case I; (**c**) the third span in Case I; (**d**) the first span in Case II; (**e**) the second span in Case II; and (**f**) the third span in Case II.

The time history results of the lateral accelerations from the finite element analysis of the bridge are plotted in Figure 8. The results indicated that the employment of the MTMD system effectively reduced the lateral accelerations.

In Case 1, before the MTMD system was applied, the maximum amplitude of the lateral acceleration of the bridge was 0.299 m/s^2^, which was reduced to 0.054 m/s^2^ by the use of the MTMD system. In Case 2, before the MTMD system was applied, the maximum amplitude of the lateral acceleration of the bridge was 0.381 m/s^2^, which was reduced to 0.099 m/s^2^ by the use of the MTMD system. The results indicate that the MTMD system is effective in mitigating lateral vibrations.

## 5. Conclusions

This research studies the lateral vibration of a long-span curved steel box girder pedestrian bridge with a small radius (20 m), and the vibration control of the bridge with MTMD. The vibration characteristics were investigated through the in-situ measurement of accelerations of the bridge and by three-dimensional finite element analysis. The lateral vibration was evaluated using ISO specifications and mitigated using MTMD. The design parameters of MTMD were evaluated through parametric studies and strategically configured to minimize the lateral vibration. The tested parameters include the mass ratio, bandwidth, center frequency ratio, and damper number. The selected deployment scheme of MTMD was evaluated through in-situ pedestrian load tests. Based on the above investigations, the following conclusions are drawn:

1. The curved steel box girder pedestrian bridge had low natural frequencies and was subjected to significant lateral vibrations under pedestrian excitation. The fundamental natural frequency of the bridge was 0.739 Hz, which is comparable with the frequencies of pedestrian loads and thus may cause resonance. Under crowd pedestrian loads, when the pace frequency is 1.8 Hz, which represents normal walking, the amplitude of lateral acceleration of the bridge exceeds the limit of ISO specifications.
2. The amplitude of lateral acceleration was greatly reduced by the use of either the STMD or MTMD. Both the STMD and MTMD reduced the amplitude to acceptable levels. Compared with the STMD, MTMD showed greater effects on reducing the lateral accelerations. When the STMD is used, the percentages of reduction are higher than 34%. When the MTMD are used, the percentages of reduction are higher than 77%. Another finding was that MTMD achieved comparable percentages of reduction at the different sections of the bridge, while the STMD led to different percentages of reduction at the different sections.
3. The effects of the mass ratio, bandwidth, center frequency ratio, and damper number of MTMD were evaluated via parametric studies. These parameters exhibited significant effects on the performance of MTMD for lateral vibration control. The mass ratio varied from 0.01 to 0.04; the bandwidth varied from 0.05 to 0.25; the center frequency ratio varied from 0.95 to 1.05, and; the damper number varied from 1 to 9. With the investigated ranges, the lateral acceleration varied in the range of 0.027–0.051 m/s^2^ for the mass ratios; 0.027–0.061 m/s^2^ for the bandwidths; 0.027–0.069 m/s^2^ for the frequency ratios, and; 0.027–0.229 m/s^2^ for the damper numbers. The comparison of these ranges indicates that the damper number has the highest effect.
4. For the studied bridge, the recommended design parameters are as follows: total mass ratio = 0.02; bandwidth = 0.15; center frequency ratio = 1.0; and damper number = 3. It should be noted that the optimal parameters are dependent on the specific bridge, while it is envisioned that the proposed step-by-step method can be applied to the design and deployment of MTMD for other bridges.
5. The performance of the optimal deployment scheme of MTMD was evaluated through in-situ pedestrian load tests in two different cases. The test results indicated that the MTMD system was effective in mitigating lateral vibrations of the bridge. The reduction percentage was in the range of 74% to 82%.

This research shows that it is important to consider the lateral vibration of curved pedestrian bridges due to the coupling effect of torsion and bending, particularly, for bridge girders with a large curvature due to the small radius. It is interesting and important to investigate the impact of bridge vibrations on durability, such as the fatigue life of the steel box girders as well as the effect of MTMD on the extension of service life of the bridges. It is envisioned that a life-cycle cost analysis can be performed to optimize the design of MTMD for the minimal life-cycle cost. The deployment positions of MTMD should also be considered in future optimization.

## Figures and Tables

**Table 1:** Natural frequencies and mode shapes of the first five modes.

Mode	Natural Frequency	Mode Shape	Note	Measured Frequency	Error
1	0.739 Hz	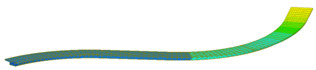	Lateral bending	0.776 Hz	5.0%
2	1.070 Hz	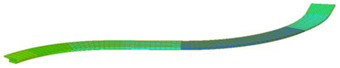	Lateral bending	1.100 Hz	2.7%
3	1.308 Hz	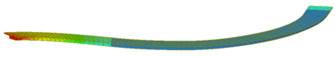	Vertical bending	1.335 Hz	2.0%
4	1.870 Hz	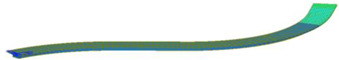	Lateral bending	1.922 Hz	2.7%
5	2.674 Hz	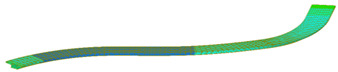	Vertical bending	2.732 Hz	2.3%

**Table 2:** Investigated pedestrian load cases.

Case	Load Type	Pace Frequency (Hz)	Pedestrian Load
1	Single pedestrian	0.739	F(t)=75sin(4.641t)
2	Single pedestrian	1.800	F(t)=75sin(11.310t)
3	Single pedestrian	2.300	F(t)=75sin(14.451t)
4	Crowd load	0.739	F(t)=961sin(4.641t)
5	Crowd load	1.800	F(t)=961sin(11.310t)
6	Crowd load	2.300	F(t)=961sin(14.451t)

**Table 3:** Simulation results of the lateral accelerations of the bridge under pedestrian loads.

Case	Span	Section	Lateral Acceleration (m/s^2^)			
			Root Mean Square	Maximum	Limit	Evaluation
1	1	1/2	0.001	0.002	0.15	Satisfied
	2	1/4	0.002	0.004		Satisfied
	2	1/2	0.002	0.004		Satisfied
	2	3/4	0.003	0.005		Satisfied
	3	1/2	0.004	0.006		Satisfied
2	1	1/2	0.004	0.009	0.15	Satisfied
	2	1/4	0.004	0.009		Satisfied
	2	1/2	0.004	0.010		Satisfied
	2	3/4	0.005	0.011		Satisfied
	3	1/2	0.008	0.017		Satisfied
3	1	1/2	0.002	0.004	0.16	Satisfied
	2	1/4	0.001	0.004		Satisfied
	2	1/2	0.001	0.004		Satisfied
	2	3/4	0.001	0.004		Satisfied
	3	1/2	0.003	0.006		Satisfied
4	1	1/2	0.017	0.061	0.30	Satisfied
	2	1/4	0.026	0.071		Satisfied
	2	1/2	0.031	0.080		Satisfied
	2	3/4	0.037	0.091		Satisfied
	3	1/2	0.052	0.136		Satisfied
5	1	1/2	0.075	0.229	0.30	Satisfied
	2	1/4	0.069	0.192		Satisfied
	2	1/2	0.072	0.197		Satisfied
	2	3/4	0.081	0.216		Satisfied
	3	1/2	0.131	0.345		**Unsatisfied**
6	1	1/2	0.031	0.098	0.32	Satisfied
	2	1/4	0.027	0.092		Satisfied
	2	1/2	0.028	0.087		Satisfied
	2	3/4	0.031	0.098		Satisfied
	3	1/2	0.047	0.157		Satisfied

**Table 4:** Lateral acceleration results of the bridge under load case 5.

Span	Section	No Damper	STMD		MTMD	
		Value (m/s^2^)	Value (m/s^2^)	Reduction	Value (m/s^2^)	Reduction
1	1/2	0.229	0.082	64%	0.052	77%
2	1/4	0.192	0.109	43%	0.044	77%
2	1/2	0.197	0.124	37%	0.038	78%
2	3/4	0.216	0.145	22%	0.041	81%
3	1/2	0.345	0.229	34%	0.059	83%

**Table 5:** Design parameters of MTMD under different total mass ratios.

Mass Ratio	*k*_1_ (kN/m)	*k*_2_ (kN/m)	*k*_3_ (kN/m)	*m*_1_ (kg)	Total Mass (kg)
0.01	36.0	42.1	48.6	33.5	100.6
0.02	72.0	84.2	97.2	67.1	201.3
0.03	108.0	126.2	145.9	100.6	301.8
0.04	144.0	168.3	194.5	134.1	402.4

**Table 6:** Effect of the mass ratio on the lateral acceleration of the bridge under load case 5.

Span	Section	No Damper	MTMD			
			*μ* = 0.01	*μ* = 0.02	*μ* = 0.03	*μ* = 0.04
1	1/2	0.229	0.049	0.048	0.049	0.051
2	1/4	0.192	0.036	0.034	0.037	0.039
2	1/2	0.197	0.030	0.027	0.031	0.034
2	3/4	0.216	0.035	0.030	0.033	0.037
3	1/2	0.345	0.047	0.040	0.044	0.049

**Table 7:** Design parameters of MTMD under different bandwidths.

Bandwidth	*k*_1_ (kN/m)	*k*_2_ (kN/m)	*k*_3_ (kN/m)	*m*_1_ (kg)	Total Mass (kg)
0.05	80.0	84.2	88.4	67.1	201.3
0.10	75.9	84.2	92.8	67.1	201.3
0.15	72.0	84.2	92.8	67.1	201.3
0.20	68.2	84.2	101.8	67.1	201.3
0.25	64.4	84.2	106.5	67.1	201.3

**Table 8:** Effect of the bandwidth on the lateral acceleration of the bridge under load case 5.

Span	Section	No Damper	MTMD				
			*R* = 0.05	*R* = 0.10	*R* = 0.15	*R* = 0.20	*R* = 0.25
1	1/2	0.229	0.058	0.053	0.048	0.049	0.061
2	1/4	0.192	0.042	0.037	0.034	0.038	0.045
2	1/2	0.197	0.037	0.033	0.027	0.034	0.041
2	3/4	0.216	0.041	0.036	0.030	0.038	0.044
3	1/2	0.345	0.057	0.044	0.040	0.048	0.058

**Table 9:** Design parameters of MTMD under different center frequency ratios.

Center Frequency Ratio	*k*_1_ (kN/m)	*k*_2_ (kN/m)	*k*_3_ (kN/m)	*m*_1_ (kg)	Total Mass (kg)
0.05	64.4	75.9	88.4	67.1	201.3
0.10	68.9	80.8	88.4	67.1	201.3
0.15	72.0	84.2	97.2	67.1	201.3
0.20	75.2	87.6	100.9	67.1	201.3
0.25	80.0	92.8	106.5	67.1	201.3

**Table 10:** Effect of the center frequency ratio on the lateral acceleration of the bridge.

Span	Section	No Damper	MTMD				
			λ = 0.95	λ = 0.98	λ = 1.00	λ = 1.02	λ = 1.05
1	1/2	0.229	0.061	0.052	0.048	0.054	0.066
2	1/4	0.192	0.047	0.042	0.034	0.045	0.054
2	1/2	0.197	0.042	0.036	0.027	0.038	0.049
2	3/4	0.216	0.046	0.040	0.030	0.044	0.052
3	1/2	0.345	0.066	0.054	0.040	0.057	0.069

**Table 11:** Design parameters of MTMD under different damper numbers.

Number	Damper	Stiffness (kN/m)	Number	Damper	Stiffness (kN/m)
1	1	*k*_1_ = 242.4	9	1	*k*_1_ = 24.0
3	1	*k*_1_ = 72.0		2	*k*_2_ = 25.0
	2	*k*_2_ = 84.2		3	*k*_3_ = 26.0
	3	*k*_3_ = 97.2		4	*k*_4_ = 27.0
5	1	*k*_1_ = 43.2		5	*k*_5_ = 28.1
	2	*k*_2_ = 46.8		6	*k*_6_ = 27.0
	3	*k*_3_ = 50.5		7	*k*_7_ = 20.2
	4	*k*_4_ = 54.3		8	*k*_8_ = 31.3
	5	*k*_5_ = 58.3		9	*k*_9_ = 32.4

**Table 12:** Effect of the damper number on the lateral acceleration of the bridge.

Span	Section	No Damper	MTMD							
			*n* = 1		*n* = 3		*n* = 5		*n* = 9	
			Value	Mean	Value	Mean	Value	Mean	Value	Mean
1	1/2	0.229	0.082	64%	0.048	26%	0.029	17%	0.010	11%
2	1/4	0.192	0.109	43%	0.034	27%	0.040	16%	0.004	11%
2	1/2	0.197	0.124	37%	0.027	29%	0.038	16%	0.002	11%
2	3/4	0.216	0.145	33%	0.03	29%	0.026	18%	0.001	11%
3	1/2	0.345	0.229	34%	0.04	29%	0.039	18%	0.006	11%

**Table 13:** Summary of the maximum mid-span accelerations in the two cases.

Case	Description	Without MTMD			With MTMD			Reduction Percentage
		Simulation	Test	Error	Simulation	Test	Error	
I	Ten people jump at the mid-span section	0.299	0.254	15%	0.054	0.047	13%	82%
II	Ten people jump at the end-span section	0.381	0.316	17%	0.099	0.087	12%	74%

Note: 1. Error = (Simulation − Test)/Simulation × 100%. 2. Reduction percentage is based on a comparison of simulation results of the two cases.

## References

[1] Michael Stein P.E. Curved Pedestrian Bridge—Straightforward Design. Proceedings of the Structures Congress 2010.

[2] Wen Q., Hua X.G., Chen Z.Q., Yang Y., Niu H.W. (2016). Control of human-induced vibrations of a curved cable-stayed bridge: Design, implementation, and field validation. J. Bridge Eng..

[3] Živanović S., Pavic A., Reynolds P. (2005). Vibration serviceability of footbridges under human-induced excitation: A literature review. J. Sound Vib..

[4] Kasperski M. (2006). Vibration serviceability for pedestrian bridges. Proc. Inst. Civ. Eng. Struct. Build..

[5] Liu Y., Zhang Q., Bao Y., Bu Y. (2020). Fatigue behavior of orthotropic composite deck integrating steel and engineered cementitious composite. Eng. Struct..

[6] Cai Q., Zhu S. (2022). The nexus between vibration-based energy harvesting and structural vibration control: A comprehensive review. Renew. Sustain. Energy Rev..

[7] Tsipianitis A., Tsompanakis Y. (2022). Improving the seismic performance of base-isolated liquid storage tanks with supplemental linear viscous dampers. Earthq. Eng. Eng. Vib..

[8] Lu Z., Wang Z., Zhou Y., Lu X. (2018). Nonlinear dissipative devices in structural vibration control: A review. J. Sound Vib..

[9] Shen W., Niyitangamahoro A., Feng Z., Zhu H. (2019). Tuned inerter dampers for civil structures subjected to earthquake ground motions: Optimum design and seismic performance. Eng. Struct..

[10] Chen G., Bao Y. (2014). Development of Bridge Girder Movement Criteria for Accelerated Bridge Construction. U.S. Department of Transportation, Report Number NUTC R316. https://scholarsmine.mst.edu/cgi/viewcontent.cgi?article=1970&context=civarc_enveng_facwork.

[11] Farhangi V., Jahangir H., Eidgahee D.R., Karimipour A., Javan S.A.N., Hasani H., Fasihihour N., Karakouzian M. (2021). Behaviour investigation of SMA-equipped bar hysteretic dampers using machine learning techniques. Appl. Sci..

[12] Weber B., Feltrin G. (2010). Assessment of long-term behavior of tuned mass dampers by system identification. Eng. Struct..

[13] Matta E. (2018). Lifecycle cost optimization of tuned mass dampers for the seismic improvement of inelastic structures. Earthq. Eng. Struct. Dyn..

[14] Liu Y., Wang K., Mercan O., Chen H., Tan P. (2020). Experimental and numerical studies on the optimal design of tuned mass dampers for vibration control of high-rise structures. Eng. Struct..

[15] Meng F., Wan J., Xia Y., Ma Y., Yu J. (2020). A multi-degree of freedom tuned mass damper design for vibration mitigation of a suspension bridge. Appl. Sci..

[16] Cao L., Li C., Chen X. (2020). Performance of multiple tuned mass dampers-inerters for structures under harmonic ground acceleration. Smart Struct. Syst. Int. J..

[17] Nguyen X.T., Miura N., Nguyen V.T., Bui T.L. (2021). Design of multiple tuned mass damper devices and application to response control of bridge under external force. Mech. Based Des. Struct. Mach..

[18] Bai W., Dai J., Zhou H., Yang Y., Ning X. (2017). Experimental and analytical studies on multiple tuned mass dampers for seismic protection of porcelain electrical equipment. Earthq. Eng. Eng. Vib..

[19] Zuo H., Bi K., Hao H. (2017). Using multiple tuned mass dampers to control offshore wind turbine vibrations under multiple hazards. Eng. Struct..

[20] Bozer A., Özsarıyıldız Ş.S. (2018). Free parameter search of multiple tuned mass dampers by using artificial bee colony algorithm. Struct. Control. Health Monit..

[21] Yin X., Song G., Liu Y. (2019). Vibration suppression of wind/traffic/bridge coupled system using multiple pounding tuned mass dampers (MPTMD). Sensors.

[22] Zuo L., Nayfeh S.A. (2005). Optimization of the individual stiffness and damping parameters in multiple-tuned-mass-damper systems. J. Vib. Acoust..

[23] Hoang N., Warnitchai P. (2005). Design of multiple tuned mass dampers by using a numerical optimizer. Earthq. Eng. Struct. Dyn..

[24] Li H.N., Ni X.L. (2007). Optimization of non-uniformly distributed multiple tuned mass damper. J. Sound Vib..

[25] Shi W., Wang L., Lu Z. (2018). Study on self-adjustable tuned mass damper with variable mass. Struct. Control. Health Monit..

[26] Kim S.Y., Lee C.H. (2018). Optimum design of linear multiple tuned mass dampers subjected to white-noise base acceleration considering practical configurations. Eng. Struct..

[27] Poovarodom N., Kanchanosot S., Warnitchai P. (2003). Application of non-linear multiple tuned mass dampers to suppress man-induced vibrations of a pedestrian bridge. Earthq. Eng. Struct. Dyn..

[28] Li Q., Fan J., Nie J., Li Q., Chen Y. (2010). Crowd-induced random vibration of footbridge and vibration control using multiple tuned mass dampers. J. Sound Vib..

[29] Daniel Y., Lavan O., Levy R. (2012). Multiple-tuned mass dampers for multimodal control of pedestrian bridges. J. Struct. Eng..

[30] Wang D., Wu C., Zhang Y., Li S. (2019). Study on vertical vibration control of long-span steel footbridge with tuned mass dampers under pedestrian excitation. J. Constr. Steel Res..

[31] Wang C., Shi W. (2019). Optimal design and application of a multiple tuned mass damper system for an in-service footbridge. Sustainability.

[32] Lai E., Gentile C., Mulas M.G. (2017). Experimental and numerical serviceability assessment of a steel suspension footbridge. J. Constr. Steel Res..

[33] Qin S., Zhou Y.L., Kang J. (2019). Footbridge serviceability analysis: From system identification to tuned mass damper implementation. KSCE J. Civ. Eng..

[34] Chen K.L., Wang R.H., Zhang W.Z. (2020). Research on Natural Vibration Characteristics and Seismic Performance of Single-ribbed Steel Arch Bridge. IOP Conf. Ser. Mater. Sci. Eng..

[35] Blazek J. (2020). Dynamic Analysis of Footbridges as Per Eurocode. Midas Bridge. https://www.midasbridge.com/en/blog/casestudy/dynamic-analysis-of-footbridges-as-per-eurocode.

[36] International Organization for Standardization (1997). Mechanical Vibration and Shock—Evaluation of Human Exposure to Whole-Body Vibration—Part 1: General Requirements.

[37] International Organization for Standardization (2007). Bases for Design of Structures-Serviceability of Buildings and Walkways Against Vibrations.

[38] Feng P., Wang Z., Jin F., Zhu S. (2019). Vibration serviceability assessment of pedestrian bridges based on comfort level. J. Perform. Constr. Facil..

[39] Wang S.J., Lee B.H., Chuang W.C., Chang K.C. (2018). Optimum dynamic characteristic control approach for building mass damper design. Earthq. Eng. Struct. Dyn..

[40] Li C. (2002). Optimum multiple tuned mass dampers for structures under the ground acceleration based on DDMF and ADMF. Earthq. Eng. Struct. Dyn..

